# Pediatrics

**Published:** 1904-09

**Authors:** Maud J. Frye

**Affiliations:** Buffalo, N. Y.


### PROGRESS IN MEDICAL SCIENCE.

### Pediatrics.

Conducted by MAUD J. FRYE, M. D., Buffalo, N. Y.

### INFECTIONS OF THE NEWBORN.

Hamill and Nicholson (*Archives of Pediatrics,* September, 1903,) report a series of cases of hemorrhagic disease of the newborn ending fatally, in which the postmortem findings warrant the following conclusions:

*Etiology.*—Hemorrhagic conditions in the newborn may, in certain instances, be due to some of the causes formerly held accountable for all such manifestations. In the majority of instances, however, hemorrhages may be considered as symptoms of an infectious condition.

Strong evidence in favor of the infectious nature of all such conditions as melena, Winckel's disease, and the like, rests in the fact that they are observed almost exclusively in institutions and not uncommonly in epidemic form. One peculiar feature of this last observation is that bacteriological studies, while proving the cases infectious, have commonly failed to demonstrate the presence of the same microörganisms in all of the cases, a result which suggests that these infections are more often dependent upon the deficient technique of a poorly trained nurse than upon defective conditions in the ward.

The nature of the infecting organism varies widely. In the 6 cases recorded, 6 different microörganisms were isolated, viz.: the bacillus pyocyaneus, the bacillus lactis aerogenes, the colon bacillus, the staphylococcus aureus, the bacillus coli immobilis and a streptococcus. The literature contains instances of infection by the pneumococcus, Pfeiffer's bacillus, the bacillus of Babés, the bacillus hemorrhagica of Kolb, the bacillus of Gaertner, the encapsulated bacillus of Dungern, and the pneumococcus. The streptococcus, the bacillus coli commune and the staphylococcus are the ones which have been most commonly encountered.

*Origin of Infection.*—Air infections may sometimes occur. The mother's milk may be responsible for a small number of cases. A source of infection may be the bath water, but probably the most common medium is the poorly trained or careless nurse.

It is impossible to definitely fix upon the port of entry. The buccal cavity, the tonsils, pharynx and the remainder of the alimentary tract, are probably the most common; next in order the lungs. The authors believe that the cord has been given too great prominence as the point of entrance and that the other avenues named—the skin, conjunctiva, nose, ears and urogenital tract—are rarely responsible.

*Symptoms.*—The symptoms have no definite order of occurrence. Fever is usually early manifested, and may vary from a slight elevation to a hyperpyrexia. Diarrhea with malodorous, undigested, greenish, mucus stools, is present in the majority of cases. Icterus may occur and is sometimes intense. Skin eruptions of almost every description have been noted. Apathy, rapid and persistent emaciation and inability or disinclination to nurse are always noted. Hemorrhage of varying degree, from either the skin, umbilical cord, eyes, ears, nose, mouth, vagina, bladder, stomach and bowels, usually happens at some time during the course of the disease. Nervous phenomena are varied; they consist mainly of convulsions, retraction of the head, nystagmus, twitching of the muscles, tetanic and tonic spasms, strabismus, changes in the pupils, and sometimes paralysis. Cyanosis and rapid, labored and irregular respirations, usually toxic in origin, may occur early or, more frequently, late in the disease. Any decided elevation of temperature, notwithstanding the frequency of fever at this age, should arouse suspicion. It may be the only evidence of the condition for several days. In most of the cases enteritis is an early symptom, and next in order, perhaps, is the occurrence of the papular or papulovesicular eruption involving the skin of the face, neck, shoulders and forearms. If, therefore, one observes this combination—fever, greenish, mucus stools, a skin eruption of the character described, and rapid emaciation—one is justified, from the therapeutic standpoint, in considering the condition infectious. If to these manifestations are added hemorrhage, nervous phenomena, cyanosis, and rapid, irregular or labored respiration, the evidences of an infectious condition are complete. Confirmatory data and definite information as to the character of the infection can be obtained by cultures made from the blood.

*Treatment.*—Measures should be directed toward improving the general aseptic technic of maternity hospitals. The routine measure of cleaning the infant's mouth is not advisable. The nurse who has handled the bedding of a patient, treated a fissured breast, emptied a bed pan or changed a napkin, unless she be most careful in preparing her hands, will readily infect the mouth of the infant. The condition of the breasts of the mother should be carefully studied. The recognition of erosions, fissures, or any other pathological condition, should result in the immediate withdrawal of the infant. Infection once established, comparatively little can be done. Isolation in bright, sunny rooms has a good effect. The withdrawal of from 15 to 20 c.cm. of blood, immediately injecting subcutaneously 20 to 30 c.cm. of artificial serum is claimed by Delestre to have a very beneficial effect. Otherwise symptomatic treatment is to be followed.

The prognosis of these infections is distinctly bad. Mild infections do occur and, in some instances, the patients recover when the condition seems hopeless. It is a not uncommon occurrence, in private practice, to see mild cases of so-called melena recover, but in institutions the mortality from infections of any type is extraordinarily high.

THE BEST ANESTHETIC FOR CHILDREN.

Burnett (*Archives of Pediatrics,* April, 1904,) advocates the use of ether as a routine anesthetic for children except in conditions such as acute bronchitis, pneumonia, nephritis, and marked respiratory obstruction, which contraindicate its employment. The unpleasant effects of ether are due chiefly to faulty administration and the fault is in using too much ether and forcing it upon the patient too rapidly. Whatever form of inhaler is used, the ether should be greatly diluted at first and it should be increased so gradually as to avoid choking and coughing as completely as possible. In this way ether may be given to children with great satisfaction, particularly if preceded by nitrous oxid and a transition gradually made from that anesthetic to ether. Nitrous oxid should be used with great caution and sparingly in infants even in this way before ether and in all children air should be given at an early period.

In administering chloroform to a crying, struggling child it should be given at a much slower rate than during normal breathing. Children generally become quiet quite suddenly during the induction of chloroform anesthesia, and it is a wise plan to suspend the administration for a moment when this occurs and note the effect of the dose which has already been taken in. It will often be observed that a marked and prolonged effect follows the initial dose and this demonstrates the wisdom of the pause in the administration; as otherwise an unnecessary and perhaps dangerous amount may be given. When it is seen that the initial dose has not been excessive, the administration may be resumed and as the respiration is now more regular the narcosis progresses with much greater safety.

The most important principles of chloroform administration are the avoidance of a concentrated vapor and an even use of the smallest amount of the anesthetic compatible with satisfactory anesthesia. It should always be given drop by drop.

